# Synthesis and Antibacterial Activities of New Metronidazole and Imidazole Derivatives

**DOI:** 10.3390/molecules14072431

**Published:** 2009-07-08

**Authors:** Abdul Jabar Kh. Atia

**Affiliations:** Department of Chemistry, College of Sciences, The University of Mustansiriayah, Iraq; E-mail: chemabdu@yahoo.com

**Keywords:** metronidazole, biimidazole, imidazole, 1,3,4-oxadiazole, 1,2,4-triazole, 1,3,4-thiadiazole

## Abstract

New imidazole ring derivatives comprising 1,3-oxazoline, Schiff's bases, thiadiazole, oxadiazole and 1,2,4-triazole moieties are reported. 3-Aminobiimidazol-4-one compounds **7a-c** were synthesized by the reaction of compounds **6a-c** with hydrazine hydrate. Biimidazole esters **9a-c** were converted into biimidazole hydrazide esters **10a-c**. Compounds **7a-c** and **10a-c** were converted into a variety of derivatives.

## 1. Introduction

Metronidazole (MTZ, **1**) is a synthetic compound used in the treatment of infections caused by Gram negative anaerobic bacteria like *Helicobacter pylori* and protozoa such as *Giardia*, *Lamblia*, and *Entomoeba histolytica*, [1] Imidazole and its derivatives are of great significance due to their important roles in biological systems, particularly in enzymes, as proton donors and/or acceptors, coordination system ligands and the base of charge–transfer processes. Unlike pyrrole (a proton donor) and pyridine (a proton acceptor), 1*H*-imidazole has both proton donor and acceptor properties [2,3]. Imidazole functionalities have been used for complex reactions with different molecular components such as carboxylic acids to obtain liquid crystalline assemblies [4]. The imidazole nucleus appears in a number of naturally occurring products like the amino acids histidine and purines, which comprise many of the most important bases in nucleic acids. Imidazole derivatives possess a broad spectrum of pharmacological activities such as anticonvulsant [5], anti-Parkinson [6] and mono-aminooxidase (MAO) inhibitory [7] activity. Oxadiazole, triazole and thiadiazole chemistry has been developed extensively and are still being developed presently. There are a number of drugs used clinically [8] which comprise oxadiazole, triazole and thiadiazole moieties in association with various heterocyclic rings. In view of these facts, a project was undertaken to synthesize a new series of imidazoles containing oxadiazole, triazole, thiadiazole and Schiff's bases and to evaluate the new compounds for their biological activity

## 2. Results and Discussion

**Scheme 1:**
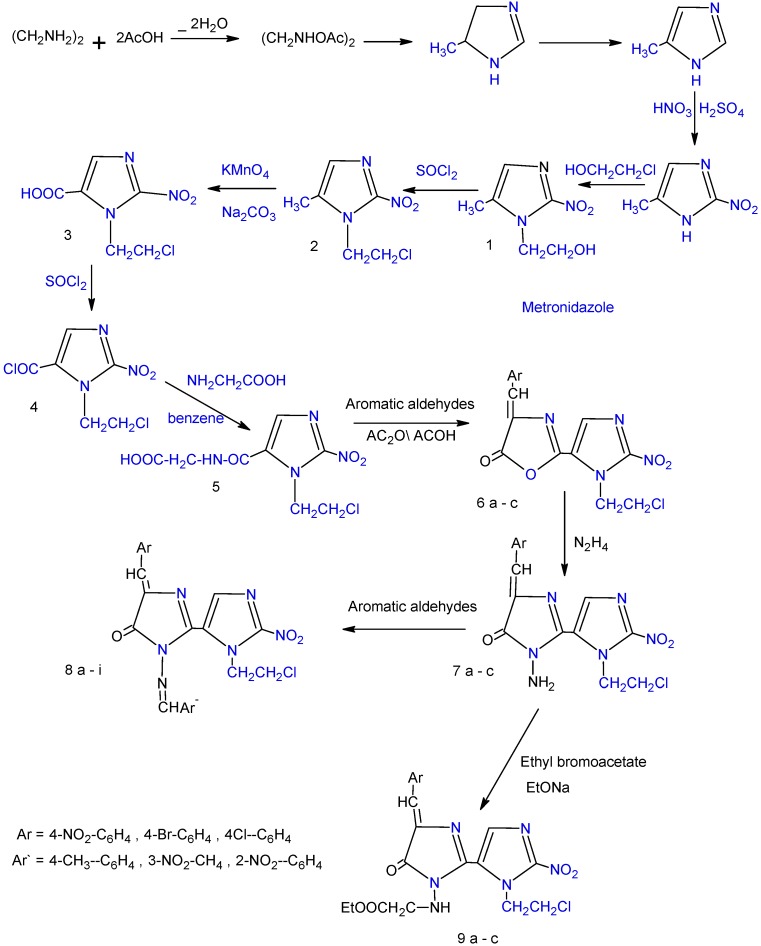
The synthesis of compounds **1 - 9a-c.**

**Scheme 2:**
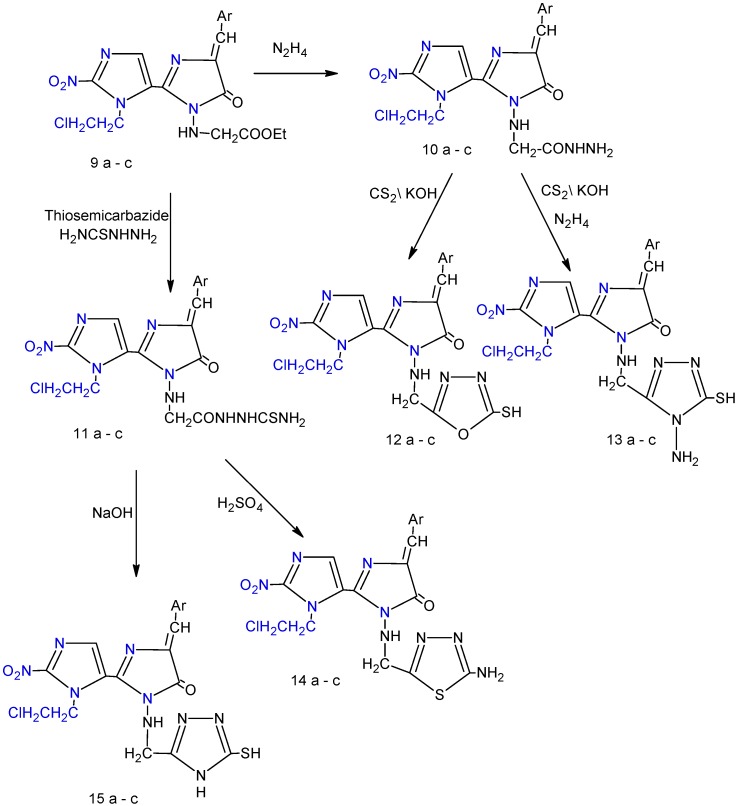
The synthesis of compounds **10(a-c) – 15(a-c)**.

The designated compounds were synthesized according to Scheme 1 and Scheme 2. Reaction of 2-(5-methyl-2-nitro-1*H*-imidazole-1-yl) ethanol (metronidazole, **1**) with thionyl chloride afforded 1-(2-chloroethyl)-5-methyl-2-nitro-1*H*-imidazole (**2**) [4]. 

The IR spectrum of the product collected and recrystallized from ethanol indicated the absence of absorption bands due to OH and the presence of a C-Cl absorption band at (768 cm^-1^). 1-(2-Chloro-ethyl)-2-nitro-1*H*-imidazol-5-carboxylic acid (**3**) [9] which was readily prepared via oxidation of the CH_3_ group of compound **2**, was converted into acid chloride **4** through reaction with thionyl chloride. The structures of compounds **3** and **4** were confirmed by ^1^H-NMR and IR spectral data and elemental analysis. In the IR spectrum compound **3** the presence of an OH absorption at 3,270- 2,650 cm^-1^ besides the C=O absorption at 1,715 cm^-1^ was observed. The ^1^H-NMR spectrum showed a triplet at 3.51-3.88 ppm integrating for protons of the CH_2_-Cl and a triplet at 2.95-3.21 ppm integrating for two protons of the N-CH_2_. The IR spectrum of compound **4** showed disappearance of the absorption band due to OH and an increase in the frequency of carbonyl moiety. Reaction of compound **4** with an amino acid (glycine) gave ({[1-(2-chloroethyl)-2-nitro-1H-imidazole-5-yl] carbonyl}amino) acetic acid (**5**) [10], while on the other hand, oxidative cyclization of compound **5** with aromatic aldehydes (Scheme 1) afforded 2-[1-(2-chloroethyl)-2-nitro-1H-imidazole-5-yl]-4-arylidene1,3-oxazol-5(4H)-ones **6a-c** [11]. The IR spectrum of compound **5 **showed two sharp absorption bands, the first appears at 1,720 cm^-1^ and is attributed to carbonyl function of the carboxylic acid and the other, observed at 1,690 cm^-1^, was assigned to a C=O stretching frequency corresponding to the amide carbonyl. In the ^1^H-NMR spectrum, the proton signals due to (CH_2_-NH) resonated at 4.42-4.67 ppm, integrating for two protons, while the proton signals due to ethyl group (N-CH_2_^a^-CH_2_^b^-Cl) were recorded between 2.82-2.93 ppm integrating for two protons (a) and at 3.40-3.64 ppm integrating for two protons (b). The structures of compounds **6a-c** were indicated by the absence of the characteristic O—H stretching in addition to the absorption bands for the NH. The ^1^H-NMR spectra of compounds **6a-c** showed new signals observed at 6.55-6.81 ppm integrating for two protons and at 7.31-7.75 ppm integrating for two protons assigned to aryl groups. The key intermediate 3-amino-3'-(2-chloroethyl)-5-arylidene-2'-nitro-3,5-dihydro-3'*H*,4*H*-2,4'-biimidazol-4-ones **7a-c** [12] were prepared from the reaction of hydrazine hydrate with compounds **6a-c**. The structures of all compounds **7a-c** were proven based on the melting point (m.p), thin layer chromatography (TLC) and spectral data. The spectra of compounds **7a-c** exhibited a NH_2_ stretching vibration at 3,360-3,210 cm^-1^ and C=O stretching vibrations at 1,660-1,695 cm^-1^. Reaction of compounds **7a-c **with aromatic aldehydes produced new Schiff's bases **8a-c **in high yield (Scheme1). The Schiff's bases **8a-i** display in their IR carbonyl and isomethine absorptions near 1,690-1,643 cm^-1^ and 1640-1627 cm^-1^, respectively, in addition to absence of NH_2_stretching vibrations. Alkylation of compounds **7a-c **with ethyl bromoacetate give ethyl {[3'-(2-chloroethyl)-4-arylidene-2'-nitro-5-oxo-4, 5-dihydro-1H, 3'H-2, 4'-biimidazol-1-yl] amino} acetates **9a-c **[13]. The formation of compounds **9a-c** was confirmed by the presence of a sharp absorption near 1,730-1,715 cm^-1^ for the ester C=O and at 1,250-1,300 cm^-1^ due to C—O stretching. In the ^1^H NMR spectra, the proton signals due to ethyl group of ester O-CH_2_^c^-CH_3_^d^ were recorded between 1.35-1.78 ppm, integrating for three protons (d) and 3.31-3.78 ppm integrating for two protons (c). The treatment of compounds **9a-c** with hydrazine hydrate, gave thiosemicarbazide compounds **10a-c** [6] and compounds **11a-c **[6], respectively. The spectral data of compounds **10a-c** and **11a-c** are given in the Experimental section. Acid hydrazides are useful intermediates leading to the formation of some heterocyclic ring such as 1,3,4-oxadiazoles, 1,3,4-thiadiazoles and 1,2,4-triazoles. The 3'-(2-chloroethyl)-5-arylidene-3-{[5-mercapto-1,3,4-oxadiazol-2-yl-methyl]amino}-2'-nitro-3,5-dihydro-3'*H*,4*H*,2,4'-biimidazol-4-one compounds **12a-c** [13] were synthesized from the reaction of compounds **10a-c** with carbon disulfide in the presence of potassium hydroxide (Scheme 2). 

The IR spectra of compounds **12a-c** displayed the SH absorption at 2,470-2,580 cm^-1^ in addition to the C=S absorption at 1,210-1,280 cm^-1^. The NH and SH protons derived from the tautomeric equilibrium resonated between 12.55-13.19 ppm as a broad singlet integrating for one proton. Moreover, NHNH_2_ signals disappeared from the ^1^H-NMR and IR spectra. The condensation of the same intermediates **10a-c** with carbon disulphide in basic media produced a potassium salt, that without isolation and purification was treated with hydrazine hydrate to give 3-{[(4-amino-5-mercapto-4*H*-1,2,4-triazol-3-yl)methyl]amino}-3'-(2-chloroethyl)-5-arylidene-2'-nitro-3',5-dihydro-3'*H*,4*H*-2,4'-biimidazol-4-ones **13a-c** [14]. In contrast to those of **12a-c**, the IR spectra of compounds **13a-c** contained additional NH_2_ absorption bands. Moreover, proton signals due to NH_2_ group of compounds **13a-c** resonated at 6.42-6.53 ppm, integrating for two protons. Oxidative cyclization of compounds **11a-c** with aqueous sodium hydroxide (Scheme 2) afforded 3'-(2-chloroethyl)-5-arylidene-2'-nitro-3-[(4*H*-1,2,4-triazol-3-yl-methyl)amino]-3,5-dihydro3'*H*,4*H*-2,4'-biimidazol-4-ones **14a-c** [7], while the treatment of the same compounds **11a-c** with conc. H_2_SO_4_ afforded 3-{[(5-amino-1,3,4-thiadiazol-2-yl) methyl]amino}-3'-(2-chloroethyl)-5-arylidene-2'-nitro-3,5-dihydro-3'*H*,4*H*-2,4'-biimidazol-4-ones **15a-c **[14].

The formation of the 1,2,4-triazole derivatives **14a-c** as confirmed by the presence of weak absorptions near 2,570-2,630 cm^-1^ for SH and 1,230-1,300 cm^-1^ due to C=S. The structures of compounds **15a-c** were confirmed by ^1^H-NMR, IR and elemental analysis. The data are shown the Experimental section.

### 2.1. Antibacterial activity

The antibacterial activity of the imidazole derivatives was tested by the agar disc-diffusion method against *Staph. aureus, E. coli and Proteus mirabilis* bacteria. Dimethylsulphoxide (DMSO) was used as solvent control, and the concentration of tested compounds was 10^-3^ M. The results of these studies are summarized in Table 1. It could be observed that all the tested compounds were active toward *Proteus mirabilis*, except compound **8b**, and all the tested compounds were active toward *E. Coli,* except for compounds **7a** and **8d** and all the compounds were active toward *Staph. aureus* except **8b, 8f, 8i, 13a**, **13b** and **14c**. On the other hand, compounds **15a-c** showed high inhibition toward all kinds of bacteria tested. In addition compounds **6a**, **8c** and **8h**, compounds **7c**, **12b**, **13c** and **14a** and compounds **7b**, **12c**, **13a-c**, **14b** and **14c** showed high inhibition toward *Staph. aureus*, *E. coli*. and *Proteus mirabilis*, respectively.

**Table 1:** Antibacterial Activity of New Compounds.

Compound No.	*Staph. aureus*	*E. coli*	*Proteus mirabilis*
DMSO	-	-	-
**6a**	+++	++	++
**6b**	+	++	++
**6c**	++	+	+
**7a**	++	-	++
**7b**	++	++	+++
**7c**	+	+++	++
**8a**	++	++	+
**8b**	-	+	-
**8c**	+++	++	++
**8d**	++	-	++
**8e**	++	+	+
**8f**	-	++	++
**8g**	++	+	+
**8h**	+++	++	++
**8i**	-	++	++
**9a**	+	+	+
**9b**	+	+	++
**9c**	++	+	+
**10a**	++	++	+
**10b**	++	++	++
**10c**	+	+	+
**11a**	+	+	++
**11b**	++	+	++
**11c**	++	+	++
**12a**	+	++	+
**12b**	+	+++	+
**12c**	++	++	+++
**13a**	-	++	+++
**13b**	-	+	+++
**13c**	+	+++	+++
**14a**	++	+++	++
**14b**	++	++	+++
**14c**	-	+	+++
**15a**	+++	+++	+++
**15b**	+++	+++	+++
**15c**	+++	++	+++

Zone diameter of growth inhibition: - = no inhibition, + = (3 – 6) mm, ++ = (7 – 10) mm and +++ = (11 – 15) mm. Conc. 10 ^-3 ^M.

## 3. Experimental

### 3.1. General

Melting points were determined in open capillary tubes on a Gallenkamp melting point apparatus and are uncorrected. The IR spectra were recorded on KBr disks, using a Perkin-Elmer 1600 series FTIR spectrometer. UV spectra were recorded on a Hitachi 2000 spectrophotometer. ^1^H-NMR spectra were recorded in DMSO-d_6 _on a Varian-Mercury 200 MHz Spectrometer. Combustion analysis was performed on a Carlo Erba 1106 elemental analyzer. Compound **1** was synthesized by a published method [15].

### 3.2. Synthesis of 1-(2-chloroethyl) -5-methyl-2-nitro-1H-imidazole *(**2**)*

Thionyl chloride (1.18 g , 0.01 mole) was added to a solution of compound **1** (1.71 g, 0.01 mole) in dry benzene (20 mL), then the reaction mixture was refluxed for 7 hrs. After evaporation, the product was collected and crystallized from ethanol-water. Yield: 90%; m.p. 85-87 °C; IR (ν, cm^-1^): 2,970-2,855 (C-H_aliph._), 1,605 (C=N), 1,530-1,470 (NO_2_), 768 (C-Cl); ^1^H-NMR: δ (ppm) 1.75 (s, CH_3_), 2.81-3.10 (t, N-CH_2_), 3.71-4.10 (t, CH_2_-Cl), 8.48 (s, 1H, imidazole); Anal. % calc./found for C_6_H_8_N_3_O_2_Cl (m.w. 189.5) C, 38.01/39.67; H, 4.25/4.34, N 22.16/23.34.

### 3.3. Synthesis 1-(2-chloroethyl)-2-nitro-1H-imidazol-5-carboxylic acid *(**3**)*

Compound **2** (1.89 g, 0.01 mole) was added to a solution of sodium bicarbonate (1.06 g, 0.01 mole) and potassium permanganate (1.57 g, 0.01 mole) in water (20 mL), then the reaction mixture was refluxed for 15 hrs. The reaction mixture was cooled and acidified with conc. HCl and the product was collected and recrystallized from ethanol. Yield: 55%; m.p. 181-183°C; IR (ν, cm^-1^): 3,270-2,650 (OH_acid_), 2,985-2,890 (C-H_aliph._), 1,715 (C=O_acid_), 1,615 (C=N), 1,530-1,370 (NO_2_); ^1^H-NMR: δ (ppm) 2.95-3.11 (t, N-CH_2_), 3.51-3.88 (t, CH_2_-Cl), 12.32 (s, acid OH), 8.82 (s, 1H, imidazole); Anal. % calc./found for C_6_H_6_N_3_O_4_Cl (m.w. 219.5): C, 32.82/33.65; H, 2.75/3.32; N, 19.14/18.09.

### 3.4. Synthesis of 1-(2-chloroethyl)-2-nitro-1H-imidazole-5-carbonyl chloride *(**4**)*

This compound was synthesized following the same procedure used in synthesis of compound **2**, without purification. Yield: 83%; m.p. 127-129°C; IR (ν, cm^-1^): 2,988-2,850 (C-H_aliph._), 1,765 (C=O_acid chloride_), 1620 (C=N), 1570-1390 (NO_2_); ^1^H-NMR: δ (ppm) 2.73-2.91 (t, N-CH_2_), 3.75-3.95 (t, CH_2_-Cl) 8.75 (s, 1H, imidazole); Anal. % calc./found for C_6_H_5_N_3_O_3_Cl_2_ (m.w. 238): C, 30.28/29.69; H, 2.12/2.79; N, 17.65/18.07.

### 3.5. Synthesis of ({[1-(2-chloroethyl)-2-nitro-1H-imidazole-5-yl] carbonyl}amino) acetic acid *(**5**)*

Compound **4** (2.38 g, 0.01 mole) was added to a stirring solution of glycine (0.75 g, 0.01 mole) and sodium hydroxide (10 mL, 10% solution). Then, the reaction mixture was shaken vigorously for 1 hr, and a few grams of crushed ice were added with stirring. After that, the solution was acidified with conc. HCl and the product was collected and recrystallized from ethanol. Yield: 80%; m.p. 165-167°C; IR (ν, cm^-1^): 3,220 (NH), 3,150 (OH_acid_), 2,985-2,870 (C-H_aliph._), 1,720 (C=O_acid_), 1,690 (C=O_amide_), 1,510-1,370 (NO_2_); ^1^H-NMR: δ (ppm) 2.82-2.93 (t, N-CH_2_), 3.40-3.72 (t, CH_2_-Cl), 4.42-4.66 (s, CO-CH_2_-NH), 8.72 (s, 1H, imidazole), 10.57 (s, NH_amide_), 12.20 (s, OH_acid_); Anal. % calc./found for C_8_H_9_N_4_O_5_Cl (m.w. 276.5): C, 34.73/35.54; H, 3.26/3.55; N, 20.25/21.28. 

### 3.6. Synthesis of 2-[1-(2-chloroethyl)-2-nitro-1H-imidazole-5-yl]-4-arylidene1,3-oxazol-5(4H)-ones ***6a-c***

Aromatic aldehyde (0.01 mole) was added to a stirring mixture of compound **5** (2.76 g, 0.01 mole) acetic acid (5 mL) and acetic anhydride (20 mL). The temperature of reaction was increased to 70 °C for 10 min., then the mixture was poured into crushed ice and stirred for 30 min. the product was collected and recrystallized from ethanol to afforded the desired compound.

*2-[1-(2-Chloroethyl)-2-nitro-1H-imidazole-5-yl]-4-(4-nitrophenyl)1,3-oxazol-5(4H)-one* (**6a**): Yield: 53%; m.p. 201-204 °C; IR (ν, cm^-1^): 3,050 (C-H_ar._), 2,983-2,868 (C-H_aliph._), 1,710 (C=O_oxazole_), 1,620 (C=C_alkene_), 1,280 (C-O); ^1^H-NMR: δ (ppm) 2.50-2.71 (t, N-CH_2_), 3.11 (s, C=CH-), 3.42-363 (t, CH_2_-Cl), 6.65-6.81 (d, 2H, ArH), 7.43-775 (d, 2H, ArH), 8.72 (s, 1H, imidazole); Anal. % calc./found for C_15_H_10_N_5_O_6_Cl (m.w. 389.5): C, 45.99/45.08; H, 2.57/3.11; N, 17.88/18.79.

*2-[1-(2-Chloroethyl)-2-nitro-1H-imidazole-5-yl]-4-(4-bromophenyl)1,3-oxazol-5(4H)-one* (**6b**): Yield: **57%; **m.p. 230-232 °C; IR (ν, cm^-1^): 3,080 (C-H_ar._), 2,990-2,890 (C-H_aliph._), 1705 (C=O_oxazole_), 1,610 (C=C_alkene_), 1,280 (C-O);^ 1^H-NMR: δ (ppm) 2.33-2.56 (t, N-CH_2_), 3.21 (s, C=CH-), 3.59-3.72 (t, CH_2_-Cl), 6.55-6.71 (d, 2H, ArH), 7.31-7.59 (d, 2H, ArH), 8.85 (s, 1H, imidazole); Anal. % calc./found for C_15_H_10_N_4_O_4_BrCl (m.w. 425.5): C, 42.33/43.64; H, 2.37/3.00; N, 13.16/14.23.

*2-[1-(2-Chloroethyl)-2-nitro-1H-imidazole-5-yl]-4-(4-chlorophenyl1,3-oxazol-5(4H)-ones* (**6c**): Yield: 51%; m.p. 236-239 °C; IR (ν, cm^-1^): 3,050 (C-H_ar._), 2,896-2,810 (C-H_aliph._), 1,725 (C=O_oxazole_), 1,610 (C=C_alkene_), 1,300 (C-O);^ 1^H-NMR: δ (ppm) 2.11-2.31 (t, N-CH_2_), 2.92 (s, C=CH-), 3.40-3.61 (t, CH_2_-Cl), 6.75-6.85 (d, 2H, ArH), 7.39-7.50 (d, 2H, ArH), 8.63 (s, 1H, imidazole); Anal. % calc./found for C_15_H_10_N_4_O_2_Cl_2_ (m.w. 381.5): C, 47.27/47.44; H, 2.64/2.89; N, 14.70/15.06.

### 3.7. Synthesis of 3-amino-3'-(2-chloroethyl)-5-arylidene-2'-nitro-3,5-dihydro-3'H,4H-2,4'biimidazol-4-ones ***7a-c***

Hydrazine hydrate (99%, 10 mL) was added to a mixture of compound 6 (0.01 mole) in dry pyridine (5 mL). The reaction mixture was refluxed for 20 hrs. Then, the mixture was allowed to cool to room temperature and pyridine was removed. The product was recrystallized from ethanol to afford the desired compound.

*3-Amino-3*'*-(2-chloroethyl)-5-(4-nitrophenyl)-2*'*-nitro-3,5-dihydro-3*'*H,4H-2,4*'*biimidazol-4-one* (**7a**): Yield: 47%; m.p. 198-201°C; IR (ν, cm^-1^): 3,360-3,290 (NH_2_), 3,080 (C-H_ar._), 2,950-2,890 (C-H_aliph._), 1,695 (C=O) 1,620 (C=C_alkene_); ^1^H-NMR: δ (ppm) 2.37-2.49 ( t, N-CH_2_), 3.13 (s, C=CH-), 3.41-3.53 (t, CH_2_-Cl), 6.40 (s, NH_2_), 6.73-6.91 (d, 2H, ArH), 7.37-7.72 (d, 2H, ArH), 8.49 (s, 1H, imidazole); Anal. % calc./found for C_15_H_12_N_7_O_5_Cl (m.w. 405.5): C, 44.40/46.53; H, 2.98/3.67; N, 24.16/24.68.

*3-Amino-3*'*-(2-chloroethyl)-5-(4-bromophenyl)-2*'*-nitro-3,5-dihydro-3*'*H,4H-2,4*'*biimidazol-4-one* (**7b**): Yield: 35%; m.p. 256-259°C; IR (ν, cm^-1^): 3,310-3,260 (NH_2_), 3,060 (C-H_ar._), 2,975-2,859 (C-H_aliph._), 1,670 (C=O), 1,615 (C=C_alkene_); ^1^H-NMR: δ (ppm) 2.25-2.43 (t, N-CH_2_), 3.35 (s, C=CH-), 3.49-3.61 (t, CH_2_-Cl), 6.21 (s, NH_2_), 6.63-6.89 (d, 2H, ArH), 7.22-7.45 (d, 2H, ArH), 8.80 (s, 1H, imidazole); Anal. % calc./found for C_15_H_12_N_6_O_3_BrCl (m.w. 439.5): C, 40.98/41.79; H, 2.75/3.43; N, 19.12/20.21.

*3-Amino-3*'*-(2-chloroethyl)-5-(4-chlorophenyl)-2*'*-nitro-3,5-dihydro-3*'*H,4H-2,4*'*biimidazol-4-one* (**7c**): Yield: 38%; m.p. 283-285°C; IR (ν, cm^-1^): 3,345-3,250 (NH_2_), 3,080 (C-H_ar._), 2,990-2,890 (C-H_aliph._), 1,660 (C=O), 1,610 (C=C_alkene_);^ 1^H-NMR: δ (ppm) 2.19-2.31 (t, N-CH_2_), 3.31 (s, C=CH-), 3.52-3.73 (t, CH_2_-Cl), 6.11 (s, NH_2_), 6.61-6.85 (d, 2H, ArH), 7.42-7.69 (d, 2H, ArH), 8.74 (s, 1H, imidazole); Anal. % calc./found for C_15_H_12_N_6_O_3_Cl_2_ (m.w. 395): C, 45.59/45.03; H, 3.06/3.86; N, 21.27/22.76. 

### 3.8. Synthesis of (5Z)-3́'-(2-chloroethyl)-5-arylidene-3-(arylideneamino)-2'-nitro-3,5-dihydro-3'H,4H-2,4'-biimidazol-4-ones ***8a–i***

The corresponding aryl aldehyde (0.01 mole) was added to a stirred solution of compound 7 (0.01 mole) in absolute ethanol (20 mL) and the mixture was refluxed for 2 hrs. After cooling, the mixture was filtered and the solid recrystallized from ethanol to afford the desired compound.

*(5Z)-3́*'*-(2-Chloroethyl)-5-(4-nitrophenyl)-3-({4*'*-methylphenyl}amino)-2*'*-nitro-3,5-dihydro-3*'*H,4H-2,4*'*-biimidazol-4-one* (**8a**): Yield: 70%; m.p. 260-263°C; IR: (ν, cm^-1^) 3,080 (C-H_ar_), 2,970-2,880 (C-H_aliph._), 1,685 (C=O), 1,640 (C=N), 1,610 (C=C_alkene_); ^1^H-NMR: δ (ppm) 1.59 (s, CH_3_), 2.31-2.47 (t, N-CH_2_), 3.25 (s, C=CH-), 3.49-3.65 (t, CH_2_-Cl) 6.30-6.82 (d, 4H, ArH), 7.33-7.65 (d, 4H, ArH), 8.21 (s, N=CH-), 8.81 (s, 1H, imidazole); Anal. % calc. for C_23_H_18_N_7_O_5_Cl (m.w. 507.5): C, 54.39/55.43; H, 3.57/4.98; N, 19.30/20.20.

*(5Z)-3́*'*-(2-Chloroethyl)-5-(4-nitrophenyl)-3-({3*'*-nitrophenyl}amino)-2*'*-nitro-3,5-dihydro-3*'*H,4H-2,4*'*-biimidazol-4-one* (**8b**): Yield: 78%; m.p. 267-269°C;IR: (ν, m^-1^) 3,060 (C-H_ar._), 2,985-2,880 (C-H_aliph._), 1,690 (C=O), 1,633 (C=N), 1,612 (C=C_alkene_); ^1^H-NMR: δ (ppm) 2.22-2.49 (t, N=CH_2_), 3.19 (s, C=CH-), 3.40-3.57(t, CH_2_-Cl), 6.48-6.80 (d, 4H, ArH), 7.29-7.72 (d, 4H, ArH), 8.51 (s, N=CH-), 8.88 (s, 1H, imidazole); Anal. % calc./found for C_22_H_15_N_8_O_7_Cl (m.w. 538.5): C, 49.04/51.09; H, 2.81/3.29; N, 20.79/21.39.

*(5Z)-3́*'*-(2-Chloroethyl)-5-(4-nitrophenyl)-3-({2*'*-nitrophenyl}amino)-2*'*-nitro-3,5-dihydro-3*'*H,4H-2,4*'*-biimidazol-4-one* (**8c**): Yield: 83%; m.p. 294-295°C; IR: (ν, cm^-1^) 3,080 (C-H_ar._), 2,990-2,865 (C-H_aliph._), 1,665 (C=O), 1,628 (C=N), 1,615 (C=C_alkene_); ^1^H-NMR: δ (ppm) 2.38-2.46 (t, N=CH_2_), 3.21 (s, C=CH-), 3.42-3.60 (t, CH_2_-Cl), 6.38-6.81 (d, 4H, ArH), 7.22-7.82 (d, 4H, ArH), 8.31 (s, N=CH-), 8.92 (s, 1H, imidazole); Anal. % calc./found for C_22_H_15_N_8_O_7_Cl (m.w. 538.5): C, 49.04/50.23; H, 2.81/3.65; N, 20.79/20.87.

*(5Z)-3́*'*-(2-Chloroethyl)-5-(4-bromophenyl)-3-({4*'*-methylphenyl}amino)-2*'*-nitro-3,5-dihydro-3*'*H,4H-2,4*'*-biimidazol-4-one* (**8d**): Yield: 67%; m.p. 274-277°C; IR (ν, cm^-1^) 3,030 (C-H_ar._), 2,983-2,870 (C-H_aliph._), 1,677 (C=O), 1,632 (C=N), 1,608 (C=C_alkene_); ^1^H-NMR: δ (ppm) 1.67 (s, CH_3_), 2,32-2.52 (t, N-CH_2_), 3.35 (s, C=CH-), 3.53-3.79 (t, CH_2_-Cl), 6.22-6.75 (d, 4H, ArH), 7.31-7.73 (d, 4H, ArH), 8.18 (s, N=CH-), 8.79 (s, 1H, imidazole); Anal. % calc./found for C_23_H_18_N_6_O_3_BrCl (m.w. 541.5): C, 50.99/51.45; H, 3.35/4.02; N, 15.51/16.19.

*(5Z)-3́*'*-(2-Chloroethyl)-5-(4-bromophenyl)-3-({3*'*-nitrophenyl}amino)-2*'*-nitro-3,5-dihydro-3*'*H,4H-2,4*'*-biimidazol-4-one* (**8e**): Yield: 75%; m.p. 300 °C (dec.); IR: (ν, cm^-1^) 3,050 (C-H_ar._), 2,990-2,895 (C-H_alIph._), 1,685 (C=O), 1,640 (C=N), 1,612 (C=C_alkene_);^ 1^H-NMR: δ (ppm) 2.28-2.47 (t, N-CH_2_), 3.18 (s, C=CH-), 3.42-3.69 (t, CH_2_-Cl), 6.37-6.82 (d, 4H, ArH), 7.45-7.81 (d, 4H, ArH), 8.33 (s, N=CH-), 8.81 (s, 1H, imidazole); Anal. % calc./found for C_22_H_15_N_7_O_5_BrCl (m.w. 572.5): C, 46.13/46.86; H, 2.64/3.75; N, 17.12/18.58.

*(5Z)-3́*'*-(2-Chloroethyl)-5-(4-bromophenyl)-3-({2*'*-nitrophenyl}amino)-2*'*-nitro-3,5-dihydro-3*'*H,4H-2,4*'*-biimidazol-4-one* (**8f**): Yield: 71%; m.p. 275-278°C; IR: (ν, cm^-1^) 3,060 (C-H_ar._), 2,986-2,795 (C-H_aliph_.), 1,660 (C=O), 1,627 (C=N), 1,607 (C=C_alkene_); ^1^H-NMR: δ (ppm) 2.33-252 (t, N-CH_2_), 3.17 (s, C=CH-), 3.42-3.71 (t, CH_2_-Cl), 6.62-6.92 (d, 4H, ArH), 7.32-7.61 (d, 4H, ArH), 8.25 (s, N=CH-), 8.69 (s, 1H, imidazole); Anal. % calc./found for C_22_H_15_N_7_O_5_BrCl (m.w. 572.5): C, 46.13/44.68; H, 2.64/2.07; N, 17.12/16.65.

*(5Z)-3́*'*-(2-Chloroethyl)-5-(4-chlorophenyl)-3-({4*'*-methylphenyl}amino)-2*'*-nitro-3,5-dihydro-3*'*H,4H-2,4*'*-biimidazol-4-one* (**8g**): Yield: 68%; m.p. 291-293°C; IR: (ν, cm^-1^) 3,055 (C-H_ar._), 2,978-2,865 (C-H_aliph._), 1,655 (C=O), 1,631 (C=N), 1,610 (C=C_alkene_); ^1^H-NMR: δ (ppm) 1.34 (s, CH_3_), 2.31-2.49 (N=CH_2_), 3.21 (s, C=CH-), 3.45-3.83 (t, CH_2_-Cl), 6.58-6.83 (d, 4H, ArH), 7.35-7.85 (d, 4H, ArH), 8.29 (s, N=CH-), 8.67 (s, 1H, imidazole); Anal. % calc./found for C_23_H_18_N_6_O_3_Cl_2_ (m.w. 497): C, 55.55/55.79; H, 3.65/3.90; N, 16.90/16.84.

*(5Z)-3́*'*-(2-Chloroethyl)-5-(4-chlorophenyl)-3-({3*'*-nitrophenyl}amino)-2*'*-nitro-3,5-dihydro-3*'*H,4H-2,4*'*-biimidazol-4-one* (**8h**): Yield: 70%; m.p. 250-253°C; IR: (ν, cm^-1^) 3,063 (C-H_ar._), 2,990-2,895 (C*-*H_aliph._), 1,643 (C=O), 1,633 (C=N), 1,617 (C=C_alkene_); ^1^H-NMR: δ (ppm) 2.36-256 (t, N-CH_2_), 3.37 (s, C=CH-), 3.47-3.88 (t, CH_2_-Cl), 6.32-678 (d, 4H ArH),7.33-780 (d, 4H, ArH), 8.23 (s, N=CH-), 8.58 (s, 1H, imidazole); Anal. % calc./found for C_22_H_15_N_7_O_3_Cl_2_ (m.w. 528): C, 50.02/52.07; H, 2.86/3.83; N, 18.56/19.78.

*(5Z)-3́*'*-(2-Chloroethyl)-5-(4-chlorophenyl)-3-({2*'*-nitrophenyl}amino)-2*'*-nitro-3,5-dihydro-3*'*H,4H-2,4*'*-biimidazol-4-one* (**8i**): Yield: 86%; m.p. 303 °C (dec.); IR: (ν, cm^-1^) 3,057 (C-H_ar._), 2,977-2,863 (C-H_aliph._), 1,650 (C=O), 1,629 (C=N), 1,620 (C=C_alkene_); ^1^H-NMR: δ (ppm) 2.39-265 (t, N-CH_2_), 3.40 (s, C=CH-), 3.59-3.96 (t, CH_2_-Cl), 6.39-6.83 (d, 4H, ArH), 7.32-7.85 (d, 4H ArH), 8.27 (s, N=CH-), 8.89 (s, 1H, imidazole); Anal. % calc./found for C_22_H_15_N_7_O_3_Cl_2_ (m.w. 528): C, 50.02/51.98; H, 2.86/3.08; N, 18.56/19.21.

### 3.9. Synthesis of ethyl {[3'-(2-chloroethyl)-4-arylidene-2'-nitro-5-oxo-4,5-dihydro-1H,3'H-2,4'-biimidazol-1-yl]amino}acetates ***9a-c***

The corresponding compound **7** (0.01 mole) was refluxed with an equivalent amount of sodium in absolute ethanol for 2 hrs. Then, ethyl bromoacetate (1.81 g, 0.01 mole) was added and refluxed for an additional 5 hrs. After evaporating the solvent under reduced pressure, a solid appeared that was recrystallized from ethanol to afford the desired compound.

*Ethyl {[3'-(2-chloroethyl)-4-(4-nitrophenyl)-2'-nitro-5-oxo-4,5-dihydro-1H,3'H-2,4'-biimidazol-1-yl]-amino}acetate* (**9a**): Yield: 56%; m.p. 245-248°C; IR: (ν, cm^-1^) 3,250 (NH), 3,070 (C-H_ar._), 2,970-2,860 (C-H_aliph._), 1,727 (C=O_ester_), 1,680 (C=O_imidazole_), 1,610 (C=C_alkene_) 1,270 (C-O); ^1^H-NMR: δ (ppm) 1.35-152 (t, CH_3_-CH_2_-), 2.31-2.52 (t, N-CH_2_), 3.10 (s, C=CH), 3.35-3.49 (q, CH_2_-CH_3_), 3.65-3.92 (t, CH_2_-Cl), 5.22 (s, N-CH_2_-CO), 6.63-6.88 (d, 2H, ArH), 7.41-7.62 (d, 2H, ArH), 8.53 (s, 1H, imidazole), 10.72 (s, NH); Anal. % calc./found for C_19_H_18_N_7_O_7_Cl (m.w. 491.5): C, 46.40/45.97; H, 3.96/3.65; N,19.93/18.05. 

*Ethyl {[3'-(2-chloroethyl)-4-(4-bromophenyl)-2'-nitro-5-oxo-4,5-dihydro-1H,3'H-2,4'-biimidazol-1-yl]-amino}acetate* (**9b**): Yield: 66%; m.p. 210-212°C; IR: (ν, cm^-1^) 3,210 (NH), 3,080 (C-H_ar._), 2,966-2,890 (C-H_aliph._), 1,715 (C=O_ester_), 1,656 (C=O_imidazole_), 1,612 (C=C_alkene_), 1,300 (C-O); ^1^H-NMR: δ (ppm) 1.41-166 (t, CH_3_-CH_2_-), 2.36-2.48 (t, N-CH_2_), 3.02 (s, C=CH), 3.59-3.78 (q, CH_2_-CH_3_), 3.81-4.05 (t, CH_2_-Cl), 5.53 (s, N-CH_2_-CO), 6.71-6.93 (d, 2H, ArH), 7.37-7.52 (d, 2H, ArH), 8.69 (s, 1H, imidazole), 10.59 (s, NH); Anal. % calc./found for C_19_H_18_N_6_O_5_BrCl (m.w. 525): C, 43.41/43.67; H, 3.45/4.32; N, 15.99/15.11.

*Ethyl {[3'-(2-chloroethyl)-4-(4-chlorophenyl)-2'-nitro-5-oxo-4,5-dihydro-1H,3'H-2,4'-biimidazol-1-yl]-amino}acetate* (**9c**): Yield: 65%; m.p. 189-192°C; IR: (ν, cm^-1^) 3,205 (NH), 3,080 (C-H_ar._), 2,979-2,885 (C-H_aliph._), 1,730 (C=O_ester_), 1,670 (C=O_imidazole_), 1,620 (C=C_alkene_) 1,250 (C-O); ^1^H-NMR: δ (ppm) 1.52-178 (t, CH_3_-CH_2_-), 2.39-2.50 (t, N-CH_2_), 3.15 (s, C=CH), 3.31-3.60 (q, CH_2_-CH_3_), 3.92-4.22 (t, CH_2_-Cl), 5.01 (s, N-CH_2_-CO), 6.57-6.88 (d, 2H, ArH), 7.41-7.59 (d, 2H, ArH), 8.32 (s, 1H, imidazole), 10.53 (s, NH); Anal. % calc./found for C_19_H_18_N_6_O_5_Cl_2_ (m.w. 481): C,47.42/47.97; H, 3.77/4.29;N, 17.46/17.86. 

### 3.10. Synthesis of 2-{[3'-(2-chloroethyl)-4-arylidene-2'-nitro-5-oxo-4,5-dihydro-1H,3'H-2,4'-biimidazol-1-yl]amino}acetohydrazides ***10a-c***

A mixture of compound **9** (0.01, mole) and hydrazine hydrate (99%, 0.32 g, 0.01 mole) in ethanol (25 mL) was refluxed for 8 hrs. Upon cooling the solution a solid appeared. This was recrystallized from ethanol to afford the desired compound.

*2-{[3'-(2-Chloroethyl)-4-(4-nitrophenyl)-2'-nitro-5-oxo-4,5-dihydro-1H,3'H-2,4'-biimidazol-1-yl]-amino}acetohydrazide* (**10a**): Yield: 75%; m.p. 241-244°C; IR: (ν, cm^-1^) 3,390-3,344 (NH_2_), 3,180 (NH), 3,060 (C-H_ar._), 2,950-2,880 (C-H_aliph._), 1,690 (C=O_imidazole_), 1,650 (C=O_amide_), 1,210 (C-N); ^1^H-NMR: δ (ppm) 2.45-2.70 (t, N-CH_2_), 3.21 (s, C=CH), 3.51-3.72 (t, CH_2_-Cl), 4.65 (s, N-CH_2_-CO), 6.32 (s, NH_2_), 6.81-7.02 (d, 2H, ArH), 7.62-7.83 (d, 2H, ArH), 8.81 (s, 1H, imidazole), 10.83 (s, NH), 11.32 (s, CO-NH-N); Anal. % calc./found for C_17_H_16_N_9_O_6_Cl (m.w. 477): C, 42.73/42.99; H, 3.38/4.06; N, 26.38/27.69. 

*2-{[3'-(2-Chloroethyl)-4-(4-bromophenyl)-2'-nitro-5-oxo-4,5-dihydro-1H,3'H-2,4'-biimidazol-1-yl]-amino}acetohydrazide* (**10b**): Yield: 64%; m.p. 167-169°C; IR: (ν, cm^-1^) 3,351-3,311 (NH_2_), 3,160 (NH), 3,080 (C-H_ar._), 2,987-2,880 (C-H_aliph._), 1685 (C=O_imidazole_), 1,639 (C=O_amide_), 1,230 (C-N); ^1^H-NMR: δ (ppm) 2.63-2.90 (t, N-CH_2_), 3.30 (s, C=CH), 3.62-3.83 (t, CH_2_-Cl), 4.73 (s, N-CH_2_-CO), 6.41 (s, NH_2_), 6.73-6.92 (d, 2H, ArH), 7.51-7.72 (d, 2H, ArH), 8.67 (s, 1H, imidazole), 10.92 (s, NH), 11.82 (s, CO-NH-N); Anal. % calc./found for C_17_H_16_N_8_O_4_BrCl (m.w. 511.5): C, 39.90/40.65; H, 3.15/3.95; N, 21.90/22.73.

*2-{[3'-(2-Chloroethyl)-4-(4-chlorophenyl)-2'-nitro-5-oxo-4,5-dihydro-1H,3'H-2,4'-biimidazol-1-yl]-amino}acetohydrazide* (**10c**): Yield: 68%; m.p. 179-181°C; IR: (ν, cm^-1^) 3,363-3,300 (NH_2_), 3,180 (NH), 3,075 (C-H_ar._), 2,989-2,890 (C-H_aliph._), 1,695 (C=O_imidazole_), 1,640 (C=O_amide_), 1,220 (C-N); ^1^H-NMR: δ (ppm) 2.73-2.95 (t, N-CH_2_), 3.42 (s, C=CH), 3.67-3.80 (t, CH_2_-Cl), 4.72 (s, N-CH_2_-CO), 6.52 (s, NH_2_), 6.82-7.99 (d, 2H, ArH), 7.32-7.52 (d, 2H, ArH), 8.82 (s, 1H, imidazole), 10.85 (s, NH), 11.75 (s, CO-NH-N); Anal. % calc./found for C_17_H_16_N_8_O_4_Cl_2_ (m.w. 467): C, 43.70/43.12; H, 3.95/3.32; N, 23.98/25.00.

### 3.11. Synthesis of N-[(amino-λ4-sulfanylidyne)methyl-2-{[3'-(2-chloroethyl)-4-arylidene-2'-nitro-5-oxo-4,5-dihydro-1H,3'H-2,4'-biimidazol-1-yl]amino} acetothiosemicarbazides ***11a-c***

These compounds were synthesized by the same procedure used for compounds **10a-c**.

*N-[(Amino-λ4-sulf{4-nitrophenyl})methyl-2-{[3'-(2-chloroethyl)-4-arylidene-2'-nitro-5-oxo-4,5-dihydro-1H,3'H-2,4'-biimidazol-1-yl]amino}acetothiosemicarbazide* (**11a**): Yield: 38%; m.p. 286-289°C; IR: (ν, cm^-1^) 3,390-3,355 (NH_2_), 3,280 (NH_thiosemicarbazide_), 3,170 (NH), 3,050 (C-H_ar._), 2,983-2,890 (C-H_aliph._), 1,693 (C=O_imidazole_), 1,645 (C=O_amide_), 1,270 (C=S); ^1^H-NMR: δ (ppm) 2.56-2.78 (t, N-CH_2_), 3.27 (s, C=CH), 3.53-3.72 (t, CH_2_-Cl), 4.83 (s, N-CH_2_-CO), 6.32 (s, NH_2_), 6.58-6.71 (d, 2H, ArH), 7.61-7.85 (d, 2H, ArH), 8.86 (s, 1H, imidazole), 10.45 (s, NH), 10.91 (s, N-NH-CS), 11.72 (s, CO-NH-N); Anal. % calc./found for C_18_H_17_N_10_O_6_SCl (mw. 536.5): C, 40.27/41.87; H, 3.19/3.76; N, 26.09/25.55.

*N-[(Amino-λ4-sulf{4-bromophenyl})methyl-2-{[3'-(2-chloroethyl)-4-arylidene-2'-nitro-5-oxo-4,5-dihydro-1H,3'H-2,4'-biimidazol-1-yl]amino}acetothiosemicarbazide* (**11b**): Yield: 42%; m.p. 282-284°C; IR: (ν, cm^-1^) 3,356-3,327 (NH_2_), 3,259 (NH_thiosemicarbazide_), 3,189 (NH), 3,080 (C-H_ar._), 2,987-2,890 (C-H_aliph._), 1,700 (C=O_imidazole_), 1,650 (C=O_amide_), 1,268 (C=S); ^1^H-NMR: δ (ppm) 2.42-2.67 (t, N-CH_2_), 3.31 (s, C=CH), 3.59-3.71 (t, CH_2_-Cl), 4.78 (s, N-CH_2_-CO), 6.13 (s, NH_2_), 6.42-6.68 (d, 2H, ArH), 7.53-7.72 (d, 2H, ArH), 8.65 (s, 1H, imidazole), 10.33 (s, NH), 10.75 (s, N-NH-CS), 11.23 (s, CO-NH-N); Anal. % calc./found for C_18_H_17_N_9_O_4_SBrCl (m.w. 570.5): C, 37.87/36.31; H, 3.00/3.75; N, 22.08/23.54.

*N-[(Amino-λ4-sulf{4-chlorophenyl})methyl-2-{[3'-(2-chloroethyl)-4-arylidene-2'-nitro-5-oxo-4,5-dihydro-1H,3'H-2,4'-biimidazol-1-yl]amino}acetothiosemicarbazide* (**11c**): Yield: 35%; m.p. 293-294°C; IR: (ν, cm^-1^) 3,402-3,381 (NH_2_), 3,275 (NH_thiosemicarbazide_), 3,140 (NH), 3,090 (C-H_ar._), 2,981-2,883 (C-H_aliph._), 1,683 (C=O_imidazole_), 1,639 (C=O_amide_), 1,259 (C=S); ^1^H-NMR: δ (ppm) 2.45-2.70 (t, N-CH_2_), 3.23 (s, C=CH), 3.62-3.80 (t, CH_2_-Cl), 4.86 (s, N-CH_2_-CO), 6.33 (s, NH_2_), 6.50-6.69 (d, 2H, ArH), 7.43-7.58 (d, 2H, ArH), 8.75 (s, 1H, imidazole), 10.46 (s, NH), 10.82 (s, N-NH-CS), 11.84 (s, CO-NH-N); Anal. % calc./found for C_18_H_17_N_9_O_4_SCl (m.w. 526): C, 41.07/41.58; H, 3.26/4.08; N, 23.95/23.21.

### 3.12. Synthesis of 3'-(2-chloroethyl)-5- arylidene-3-{[5-mercapto-1,3,4-oxadiazol-2-yl-methyl] amino}-2'-nitro--3,5dihydro-3'H,4H,2,4'-biimidazol-4-ones ***12a-c***

The corresponding compound **10** (0.01 mole) and CS_2_ (0.6 mL, 0.01 mole) were added to a solution of KOH (0.56 g, 0.01 mole) in ethanol (30 mL). The reaction mixture was refluxed for 3 hrs. After evaporation under reduced pressure to dryness, a solid was obtained. This was dissolved in H_2_O (200 mL) and acidified with conc. HCl. The precipitate was filtered off, washed with water and recrystallized from ethanol to afford the desired compound.

*3*'*-(2-Chloroethyl)-5-(4-nitrophenyl)-3-{[5-mercapto-1,3,4-oxadiazol-2-yl-methyl]amino}-2*'*-nitro-3,5-dihydro-3*'*H,4H,2,4*'*-biimidazol-4-one* (**12a**): Yield: 63%; m.p. 302-303°C; IR: (ν, cm^-1^) 3,220 (NH), 3,080 (C-H_ar._), 2,987-2,890 (C-H_aliph._), 2,490 (SH), 1,670 (C=O_imidazole_), 1,265 (C=S); ^1^H-NMR: δ (ppm) 2.52-2.72 (t, N-CH_2_), 3.27 (s, C=CH), 3.51-3.78 (t, CH_2_-Cl), 4.63 (s, N-CH_2_-oxadiazole), 6.45-6.62 (d, 2H, ArH), 7.39-7.60 (d, 2H, ArH), 8.89 (s, 1H, imidazole), 10.52 (s, NH), 12.55 (s, NH + SH of oxadiazole); Anal. % calc./found for C_18_H_14_N_9_O_6_Cl (m.w. 519.5): C, 41.59/41.83; H, 2.71/3.57; N, 24.25/23.61.

*3’-(2-Chloroethyl)-5-(4-bromophenyl)-3-{[5-mercapto-1,3,4-oxadiazol-2-yl-methyl]amino}-2*'*-nitro-3,5-dihydro-3*'*H,4H,2,4*'*-biimidazol-4-one* (**12b**): Yield: 65%; m.p. 295-297°C; IR: (ν, cm^-1^) 3,235 (NH), 3,085 (C-H_ar._), 2,976-2,855 (C-H_aliph._), 2,470 (SH), 1,685 (C=O_imidazole_), 1,280 (C=S); ^1^H-NMR: δ (ppm) 2.91-3.22 (t, N-CH_2_), 3.52 (s, C=CH), 3.69-3.80 (t, CH_2_-Cl), 4.51 (s, N-CH_2_-oxadiazole), 6.34-6.50 (d, 2H, ArH), 7.50-7.69 (d, 2H, ArH), 8.49 (s, 1H, imidazole), 10.66 (s, NH), 12.95 (s, NH + SH of oxadiazole); Anal. % calc./found for C_18_H_14_N_8_O_4_BrCl (m.w. 553.5): C, 39.04/38.53; H, 2.55/2.97; N, 20.23/21.78.

*3’-(2-Chloroethyl)-5- (4-chlorophenyl)-3-{[5-mercapto-1,3,4-oxadiazol-2-yl-methyl]amino}-2*'*-nitro-3,5-dihydro-3*'*H,4H,2,4*'*-biimidazol-4-one* (**12c**): Yield: 69%; m.p. 287-289°C; IR: (ν, cm^-1^) 3,215 (NH), 3,065 (C-H_ar._), 2,978-2,860 (C-H_aliph._), 2,580 (SH), 1,690 (C=O_imidazole_), 1,210 (C=S); ^1^H-NMR: δ (ppm) 2.49-2.70 (t, N-CH_2_), 3.71 (s, C=CH), 3.92-4.00 (t, CH_2_-Cl), 4.42 (s, N-CH_2_-oxadiazole), 6.46-6.70 (d, 2H, ArH), 7.45-7.65 (d, 2H, ArH), 8.12 (s, 1H, imidazole), 10.59 (s, NH), 13.91 (s, NH + SH of oxadiazole); Anal. % calc./found for C_18_H_14_N_8_O_4_SCl_2_ (m.w. 509): C, 42.45/42.65; H, 2.77/3.23; N, 22.00/23.65.

### 3.13. Synthesis of 3-{[(4-amino-5-mercapto-4H-1,2,4-triazol-3-yl)methyl]amino}-3'-(2-chloroethyl)-5-arylidene-2'-nitro-3',5-dihydro-3'H,4H-2,4'-biimidazol-4-ones ***13a-c***

The corresponding compound **10** (0.01 mole) and CS_2_ (0.6 mL, 0.01 mole) in ethanol (20 mL) were stirred for 12 hrs. Then, diethyl ether (18 mL) was added. The precipitated solid thus obtained was filtered, washed with cold diethyl ether, and without isolation and purification dissolved in water (10 mL) and hydrazine hydrate (99%, 0.34 g, 0.01 mole) was added. The reaction mixture was refluxed for 1 hr. cooled, diluted with water and acidified with acetic acid. The precipitate was filtered off, washed with water and recrystallized from ethanol to afford the desired compound.

*3-{[(4-Amino-5-mercapto-4H-1,2,4-triazol-3-yl)methyl]amino}-3*'*-(2-chloroethyl)-5-(4-nitrophenyl)-2*'*-nitro-3*'*,5-dihydro-3*'*H,4H-2,4*'*-biimidazol-4-one* (**13a**): Yield: 53%; m.p. 243-246°C; IR: (ν, cm^-1^) 3,400-3,370 (NH_2_), 3,200 (NH), 3,060 (C-H_ar._), 2,966-2,872 (C-H_aliph._), 2,460 (SH), 1,695 (C=O_imidazole_), 1,220 (C=S); ^1^H-NMR: δ (ppm) 2.51-2.70 (t, N-CH_2_), 3.38 (s, C=CH), 3.55-3.69 (t, CH_2_-Cl), 4.81 (s, N-CH_2_-triazole), 6.42 (s, NH_2_), 6.57-6.70 (d, 2H, ArH), 7.35-7.60 (d, 2H, ArH), 8.71 (s, 1H, imidazole), 10.50 (s, NH), 13.20 (s, NH + SH of triazole); Anal. % calc./found for C_18_H_16_N_11_O_5_SCl (m.w. 533.5): C, 37.90/36.54; H, 3.00/3.76; N, 27.01/27.68.

*3-{[(4-Amino-5-mercapto-4H-1,2,4-triazol-3-yl)methyl]amino}-3*'*-(2-chloroethyl)-5-(4-bromophenyl)-2*'*-nitro-3*'*,5-dihydro-3*'*H,4H-2,4*'*-biimidazol-4-one* (**13b**): Yield: 55%; m.p. 232-235°C; IR: (ν, cm^-1^) 3,480-3,300 (NH_2_), 3,205 (NH), 3,075 (C-H_ar._), 2,980-2,880 (C-H_aliph._), 2,550 (SH), 1,700 (C=O_imidazole_), 1,250 (C=S); ^1^H-NMR: δ (ppm) 2.62-280 (t, N-CH_2_), 3.45 (s, C=CH), 3.61-3.80 (t, CH_2_-Cl), 4.52 (s, N-CH_2_-triazole ), 6.53 (s, NH_2_), 6.70-6.92 (d, 2H, ArH), 7.42-7.60 (d, 2H, ArH), 8.80 (s, 1H, imidazole), 10.94 (s, NH), 13.22 (s, NH + SH of triazole); Anal. % calc./found for C_18_H_16_N_10_O_3_BrSCl (m.w. 567.7): C, 38.07/37.53; H, 2.84/3.72; N, 24.67/45.51.

*3-{[(4-Amino-5-mercapto-4H-1,2,4-triazol-3-yl)methyl]amino}-3*'*-(2-chloroethyl)-5-(4-chlorophenyl)-2*'*-nitro-3*'*,5-dihydro-3*'*H,4H-2,4*'*-biimidazol-4-one* (**13c**): Yield: 48%; m.p. 274-276°C; IR: (ν, cm^-1^) 3,410-3,360 (NH_2_), 3,190 (NH), 3,070 (C-H_ar._), 2,971-2,890 (C-H^aliph.^), 2,560 (SH), 1,700 (C=O_imidazole_), 1,270 (C=S); ^1^H-NMR: δ (ppm) 2.36-2.52 (t, N-CH_2_), 3.67 (s, C=CH), 3.81-3.96 (t, CH_2_-Cl), 4.50 (s, N-CH_2_-triazole), 6.46 (s, NH_2_), 6.62-6.89 (d, 2H, ArH), 7.35-7.58 (d, 2H, ArH), 8.62 (s, 1H, imidazole), 10.62 (s, NH), 13.21 (s, NH + SH of triazole); Anal. % calc./found for C_18_H_16_N_10_O_3_SCl (m.w. 523): C, 38.62/36.99; H, 3.06/3.25; N, 25.02/25.93.

### 3.14. Synthesis of 3'-(2-chloroethyl)-5-arylidene-2'-nitro-3-[(4H-1,2,4-triazol-3-yl-methyl )amino]-3,5-dihydro-3'H,4H-2,4'-biimidazol-4-ones ***14a-c***

A mixture of compound **11** (0.01 mole) and sodium hydroxide (0.01 mole, as 4% solution) was stirred for 4 hrs. After cooling, the solution was acidified with conc. HCl and the precipitate was filtered and recrystallized from ethanol to afford the desired compound.

*3*'*-(2-Chloroethyl)-5-(4-nitrophenyl)-2*'*-nitro-3-[(4H-1,2,4-triazol-3-yl-methyl)amino]-3,5-dihydro-3*'*H,4H-2,4*'-*biimidazol-4-one* (**14a**): Yield: 43%; m.p. 202-205°C; IR: (ν, cm^-1^) 3,300 (triazole, NH), 3,210 (NH), 3,066 (C-H_ar._), 2,982-2,877 (C-H_aliph._), 2,625 (SH), 1,696 (C=O_imidazole_), 1230 (C=S); ^1^H-NMR: δ (ppm) 2.32-2.50 (t, N-CH_2_), 3.31 (s, C=CH), 3.62-3.81 (t, CH_2_-Cl), 4.68 (s, N-CH_2_-triazole), 6.61-6.82 (d, 2H, ArH), 7.42-7.65 (d, 2H, ArH), 8.72 (s, 1H, imidazole), 10.39 (s, NH), 11.32 (s, triazole NH) 13.37 (s, NH + SH of triazole); Anal. % calc./found for C_18_H_15_N_10_O_5_SCl (m.w. 518.5): C, 41.66/42.61; H, 2.91/3.61; N, 26.99/26.13.

*3*'*-(2-Chloroethyl)-5-(4-bromophenyl)-2*'*-nitro-3-[(4H-1,2,4-triazol-3-yl-methyl)amino]-3,5-dihydro-3*'*H,4H-2,4*'-*biimidazol-4-one* (**14b**): Yield: 61%; m.p. 213-215°C; IR: (ν, cm^-1^) 3,353 (triazole, NH), 3,190 (NH), 3,082 (C-H_ar._), 2,991-2,879 (C-H_aliph._), 2,630 (SH), 1,700 (C=O_imidazole_), 1,300 (C=S); ^1^H-NMR: δ (ppm) 2.41-2.66 (t, N-CH_2_), 3.42 (s, C=CH), 3.67-3.82 (t, CH_2_-Cl), 4.65 (s, N-CH_2_-triazole), 6.41-6.60 (d, 2H, ArH), 7.32-7.60 (d, 2H, ArH), 8.80 (s, 1H, imidazole), 10.42 (s, NH), 11.49 (s, triazole NH) 13.41 (s, NH + SH of triazole); Anal. % calc./found for C_18_H_15_N_9_O_5_SBrCl (m.w. 552.5): C, 39.11/40.71; H, 2.74/3.43; N, 22.80/23.41.

*3*'*-(2-Chloroethyl)-5-(4-chlorophenyl)-2*'*-nitro-3-[(4H-1,2,4-triazol-3-yl-methyl)amino]-3,5-dihydro-3*'*H,4H-2,4*'-*biimidazol-4-one* (**14c**): Yield: 45%; m.p. 241-245°C; IR: (ν, cm^-1^) 3,300 (triazole, NH), 3254 (NH), 3,030 (C-H_ar._), 2,980-2,855 (C-H_aliph._), 2,570 (SH), 1,689 (C=O_imidazole_), 1,256 (C=S); ^1^H-NMR: δ (ppm) 2.51-2.79 (t, N-CH_2_), 3.63 (s, C=CH), 3.60-3.85 (t, CH_2_-Cl), 4.71 (s, N-CH_2_-triazole), 6.32-6.56 (d, 2H, ArH), 7.52-7.81 (d, 2H, ArH), 8.69 (s, 1H, imidazole), 10.52 (s, NH), 11.63 (s, triazole NH) 13.39 (s, NH + SH of triazole); Anal. % calc./found for C_18_H_15_N_9_O_3_SCl_2_ (m.w. 508): C, 42.53/42.00; H, 2.97/2.13; N, 24.80/45.39. 

### 3.15. Synthesis of 3-{[(5-amino-1,3,4-thiadiazol-2-yl)methyl]amino}-3'-(2-chloroethyl)-5-arylidene-2'-nitro-3,5-dihydro-3'H,4H-2,4'-biimidazol-4-ones ***15a-c***

The corresponding compound **11** (0.01 mole) was dissolved in cold conc. sulfuric acid (10 mL) and stirred at room temperature for 24 hrs. Then, the reaction mixture was poured into crushed ice and diluted with water; the precipitate was filtered, washed with water and recrystallized from ethanol to afford the desired compound.

*3-{[(5-Amino-1,3,4-thiadiazol-2-yl)methyl]amino}-3*'*-(2-chloroethyl)-5-(4-nitrophenyl-2*'*-nitro-3,5-dihydro-3*'*H,4H-2,4*'*-biimidazol-4-one* (**15a**): Yield: 69%; m.p. 263-265°C; IR: (ν, cm^-1^) 3,402-3,366 (NH_2_), 3,205 (NH), 3,065 (C-H_ar._), 2,990-2,879 (C-H_aliph._), 1688 (C=O_imidazole_); ^1^H-NMR: δ (ppm) 2.34-2.62 (t, N-CH_2_), 3.59 (s, C=CH), 3.65-3.78 (t, CH_2_-Cl), 4.82 (s, N-CH_2_-thiadiazole), 6.41 (s, NH_2_), 6.47-6.62 (d, 2H, ArH), 7.35-7.47 (d, 2H, ArH), 8.87(s, 1H, imidazole), 10.43 (s, NH); Anal. % calc./found for C_18_H_15_N_10_O_5_SCl (m.w. 518.5): C, 41.66/41.08; H, 2.91/3.86; N, 26.99/26.12.

*3-{[(5-Amino-1,3,4-thiadiazol-2-yl)methyl]amino}-3*'*-(2-chloroethyl)-5-(4-bromophenyl-2*'*-nitro-3,5-dihydro-3*'*H,4H-2,4*'*-biimidazol-4-one* (**15b**): Yield: 78%; m.p. 271-273°C; IR: (ν, cm^-1^) 3,387-3,308 (NH_2_), 3,195 (NH), 3,076 (C-H_ar._), 2,983-2,890 (C-H_aliph._), 1,703 (C=O_imidazole_); ^1^H-NMR: δ (ppm) 2.53-2.67 (t, N-CH_2_), 3.31 (s, C=CH), 3.72-3.95 (t, CH_2_-Cl), 4.63 (s, N-CH_2_-thiadiazole), 6.45 (s, NH_2_), 6.68-6.79 (d, 2H, ArH), 7.36-7.50 (d, 2H, ArH), 8.73(s, 1H, imidazole), 10.53 (s, NH); Anal. % calc./found for C_15_H_15_N_9_O_3_SBrCl (m.w. 552.5):C, 39.11/40.87; H, 2.74/2.06; N, 22.80/22.01.

*3-{[(5-Amino-1,3,4-thiadiazol-2-yl)methyl]amino}-3*'*-(2-chloroethyl)-5-(4-chlorphenyl-2*'*-nitro-3,5-dihydro-3*'*H,4H-2,4*'*-biimidazol-4-one* (**15c**): Yield: 62%; m.p. 300-302°C; IR: (ν, cm^-1^) 3,410-3,345 (NH_2_), 3,215 (NH), 3086 (C-H_ar._), 2957-2861 (C-H_aliph._), 1,702 (C=O_imidazole_); ^1^H NMR: δ (ppm) 2.56-2.70 (t, N-CH_2_), 3.43 (s, C=CH), 3.65-3.81 (t, CH_2_-Cl), 4.83 (s, N-CH_2_-thiadiazole), 6.48 (s, NH2), 6.63-6.82 (d, 2H, ArH), 7.66-7.73 (d, 2H, ArH), 8.75(s, 1H, imidazole), 10.73 (s, NH); Anal. % calc./found for C_18_H_15_N_9_O_3_SCl_2_ (m.w. 508): C, 42.53/42.99; H, 2.97/2.22; N, 24.80/23.10.
